# Weaving oral health provider perspectives to guide future dental therapy advocacy and implementation efforts

**DOI:** 10.3389/fpubh.2025.1532934

**Published:** 2025-02-12

**Authors:** Amanda J. Llaneza, Kara A. Stone, Julie Seward

**Affiliations:** ^1^Icahn School of Medicine at Mount Sinai, New York, NY, United States; ^2^Southern Plains Tribal Health Board, Oklahoma City, OK, United States

**Keywords:** dental health aide therapists, oral health, oral health workforce, American Indian/Alaska Native, medically underserved area

## Abstract

**Introduction:**

American Indian and Alaska Native (AI/AN) communities in the United States (U.S.) face greater oral healthcare inequities than other groups in the country. A solution to improving oral healthcare access for American Indian and Alaska Native communities in the United States is the integration of dental therapists (DT). However, it has been met with opposition. The goal of this study was to explore oral health provider engagement and perspectives of DT implementation in local communities.

**Methods:**

Engagement related to DT from the dental provider perspective from the ADTA conference's attendees were analyzed via optional, electronic post-conference surveys. Descriptive statistics were utilized to report the position type of attendees, years of experience in the role, incorporation of clinical courses for DT scope of practice, and clinic management and administration for their patients. Surveys included two open-ended questions inquiring about personal objectives and comments and suggestions regarding the conference overall. From attendee's open-ended statements on objectives for attending this conference, key patterns were described.

**Results:**

One hundred people nationwide attended the conference. Of these attendees, 56% completed the post-conference survey evaluation. Among the respondents, 25% identified as dentists, 23% identified as dental hygienists, 46% identified as dental therapists, 6% identified as other oral health workforce members (i.e., dental assistant and/or Tribal oral health coordinator). The majority felt the strategies covered related to DT could be incorporated regardless of the type of workforce position. Fifty-seven percent of dentists felt it would be easy or very easy to implement strategies from the conference, and 75% of dental hygienists and other oral health workforce members felt it was easy or very easy to implement strategies from the conference. Education, advocacy, and networking were identified as the main objectives for attending the conference.

**Discussion:**

The conference platform encouraged dissenting viewpoints to contribute to academic and policy debates to ultimately provide broader access to oral healthcare through a comprehensive team approach. There was strong support across positions to advocate for and expand DT to the lower 48 states of the U.S., particularly in areas with underserved communities. Additionally, most providers felt this could be done with ease and no providers indicated opposition in open-ended remarks. Collaborative efforts among policymakers, dental associations, educational institutions, and community advocates can create a pathway for the successful integration of dental therapists into the healthcare system.

## Introduction

American Indian and Alaska Native (AI/AN) communities in the United States (U.S.) are disproportionately impacted by poor oral health compared to the general population (1–3). The longstanding challenges that this population faces are related to systemic barriers to oral healthcare such as geographic isolation, socioeconomic factors, and historical and current disparities in healthcare funding and infrastructure (4–6). Research has indicated the shortage of oral health providers is a major factor that has limited access to oral healthcare for AI/AN populations (4–6).

One possible solution to improving access to care and reducing oral health disparities, especially among this population, is by expanding the oral health workforce with the introduction of dental health aide therapists, also referred to as dental therapy (DT), which is under the umbrella of the Tribal Community Health Aide Program (CHAP) (7–9). CHAP aims to integrate traditional healing practices into its educational curriculum of Health Aides, with an emphasis on culturally responsive services in the community (9). Dental Therapy serves to reduce oral health disparities in AI/AN communities by educating and training community members to provide care as Primary Dental Health Aides, Expanded Function Dental Health Aides, Dental Health Aide Hygienists, and Dental Health Aide Therapists. The scope of practice for each position ranges from delivering preventative services (e.g., providing fluoride varnish, sealants, atraumatic restorative treatments, cleanings, and assisting with nutritional counseling, oral hygiene instruction, and dental radiology) to providing dental services equivalent to a physician's assistant (e.g., developing treatment plans and continuing care in areas struggling to recruit and retain providers) (10). These providers extend culturally competent oral healthcare services to remote, rural, and often marginalized populations under the direction and supervision of a dentist and have effectively improved oral health outcomes amongst Indigenous communities of the United States, Canada, New Zealand, and 48 other countries (7, 8, 10, 11). Thus far, 26 U.S. states have authorized, or are considering authorization of DT with varying scopes of practice and educational requirements (12). Regardless of this support, DT has been met with strong opposition from dentists, and more specifically the American Dental Association, citing safety and efficacy concerns (13).

Though DT is an evidenced-based approach to improving oral health care, it is important to understand the dental provider's perspective of incorporating DT into the oral health workforce among rural and Tribal populations in the state of Oklahoma. While there is no active legislation to authorize dental therapy in Oklahoma at the time of submission of this manuscript, education and outreach efforts to provide evidence-based resources and collaborative learning opportunities are underway. Establishing common ground among members of the dental care team is paramount for the best possible outcome, which is evidence-informed policy that leads to improved access to care and improved general wellness for all. The objective of this manuscript was to begin to understand the oral health provider perspective of DT implementation in local communities, and engagement with providers who attended the American Dental Therapy Association's (ADTA) conference.

## Methods

The Southern Plains Tribal Health Board (SPTHB) is a tribally designated 501(c) (3) non-profit organization. It is an American Indian representative body based in Oklahoma and is also a federally funded Tribal Epidemiology Center. The SPTHB serves 43 Tribal Nations within the Oklahoma City Indian Health Service Area of Kansas, Oklahoma, and Texas.

SPTHB co-chaired the inaugural, in-person ADTA's Annual Conference in Oklahoma City, Oklahoma in October 2023. One of the goals of the conference was to present the benefits and potential of DT and provide actionable steps for implementation in communities across the nation. Tribal dental teams participated in state-wide discussions alongside elected officials to learn more about how dental therapy could be integrated into Tribal clinics to improve access to oral healthcare and overall wellness. The conference covered topics such as dental therapy education programs, advocacy tools for dental therapy, and the current state of dental therapy nationally and within specific states. SPTHB and co-chairs hoped to establish a balanced learning platform with a diverse range of perspectives from the oral healthcare workforce to better understand how DT can address oral health disparities within communities. Ethical approval was not obtained for this study because it is considered secondary research for which consent is not required. Identifiable private health information was not accessed.

### Analysis

Engagement related to DT from the provider perspective of those who attended the ADTA conference in Oklahoma City was analyzed using an optional, electronic survey that also offered Continuing Dental Education credits that were made available to all attendees at the conclusion of the event (Table 1). Descriptive statistics illustrated the type of position (i.e., dentist, dental hygienist, dental therapist, or other) of the attendees, and their years of job experience. Attendees also indicated on the survey their feelings regarding the incorporation of clinical courses for DT scope of practice and clinic management and administration for their patients. Attendees described if it would be very easy, easy, somewhat easy, neither easy nor difficult, somewhat difficult, difficult, or very difficult to incorporate DT into their practices. Descriptive statistics were used to portray results and created categorical variables of very easy, easy (easy and somewhat easy), neither easy nor difficult, difficult (difficult and somewhat difficult), and very difficult. Lastly, attendees had an opportunity to share their feelings about the conference using open-ended statements. Open-ended questions included, “What were your objectives for attending this conference?” and “Any further comments or suggestions?” Qualitative research methodologies for the content analysis of the open-ended responses were utilized (14, 15). The analysis included one researcher qualitatively exploring the data to develop meaningful conclusions, and two other researchers confirmed the independent review. This manuscript emphasized a deductive approach for identifying and describing key patterns (15, 16).

**Table 1:** Post conference survey questions.

Q1.	Please enter your first and last name.
Q2.	Please enter your email address.
Q3.	Do you need CE credits for the event?
Q4.	How many years have you been practicing in the oral health care industry?
Q5.	Please identify your position.
Q6.	Which programs did you attend? Select all that apply.
Q7.	Presenter specific questions:
	a. Effectiveness and organization
	b. Clear objectives
	c. Objectives met
	d. Course was worth my time
	e. Materials and audio visuals
	f. Chance of attending another course by this speaker
	g. I learned as a result of the CE program
	h. The content of the program(s) are useful for my practice or professional development
Q8.	Overall, you were satisfied with:
	a. Your educational experience
	b. The method of instructional delivery
	c. The all-inclusive registration fee for the conference
	d. The accessibility of the conference
	e. The conference venue
Q9.	How easy do you feel it would be to incorporate the strategies covered in the conference into your care of your patients and the running of your practice, healthcare clinic, etc.?
Q10.	What were your objectives for attending this conference? (open ended)
Q11.	Were your objectives met? (open ended)
Q12.	Any further comments or suggestions? (open ended)

Authors did not have direct access to the survey platform software. To maintain anonymity, the survey questions were provided from the ADTA.

## Results

One hundred people attended the conference from across the nation. Of the 100 attendees, 56 (56%) completed the post-conference survey evaluation. Response rates of open-ended questions varied with 45/56 (80%) of attendees answering, “What were your objectives for attending this conference?” and 24/56 (43%) responding to, “Any further comments or suggestions?” Of those who responded, common objectives were to gain information on dental therapy at the state and national level and how it can be utilized in their respective organizations, understand ways to advocate for dental therapy and support dental therapy initiatives, and to connect and collaborate with other supporters of dental therapy. Further comments and suggestions included expressions of gratitude and encouragement, requests for more content and variety in presentation topics, “none”/“no”/“no comments!”, increased continuing education credits and opportunity to improve skills, and comments on conference venue and break times between speakers. For participant descriptives see Table 2.

**Table 2:** Survey respondent descriptives.

		**Years of job experience**			
**Type of position**	**Total**	**0–5**	**6–15**	**16–25**	**26**+
Dentist	14 (25%)	1 (7%)	1 (7%)	8 (57%)	4 (29%)
Dental hygienist	13 (23%)	2 (15%)	4 (31%)	0 (0%)	7 (54%)
Dental therapist	26 (46%)	8 (31%)	10 (38%)	6 (23%)	2 (8%)
Other	3 (6%)	1 (33%)	0 (0%)	2 (67%)	0 (0%)

*N* = 56, all data displayed as *n* (%), Other = Other oral health workforce members (i.e., dental assistant and/or Tribal oral health coordinator).

**Figure 1:**
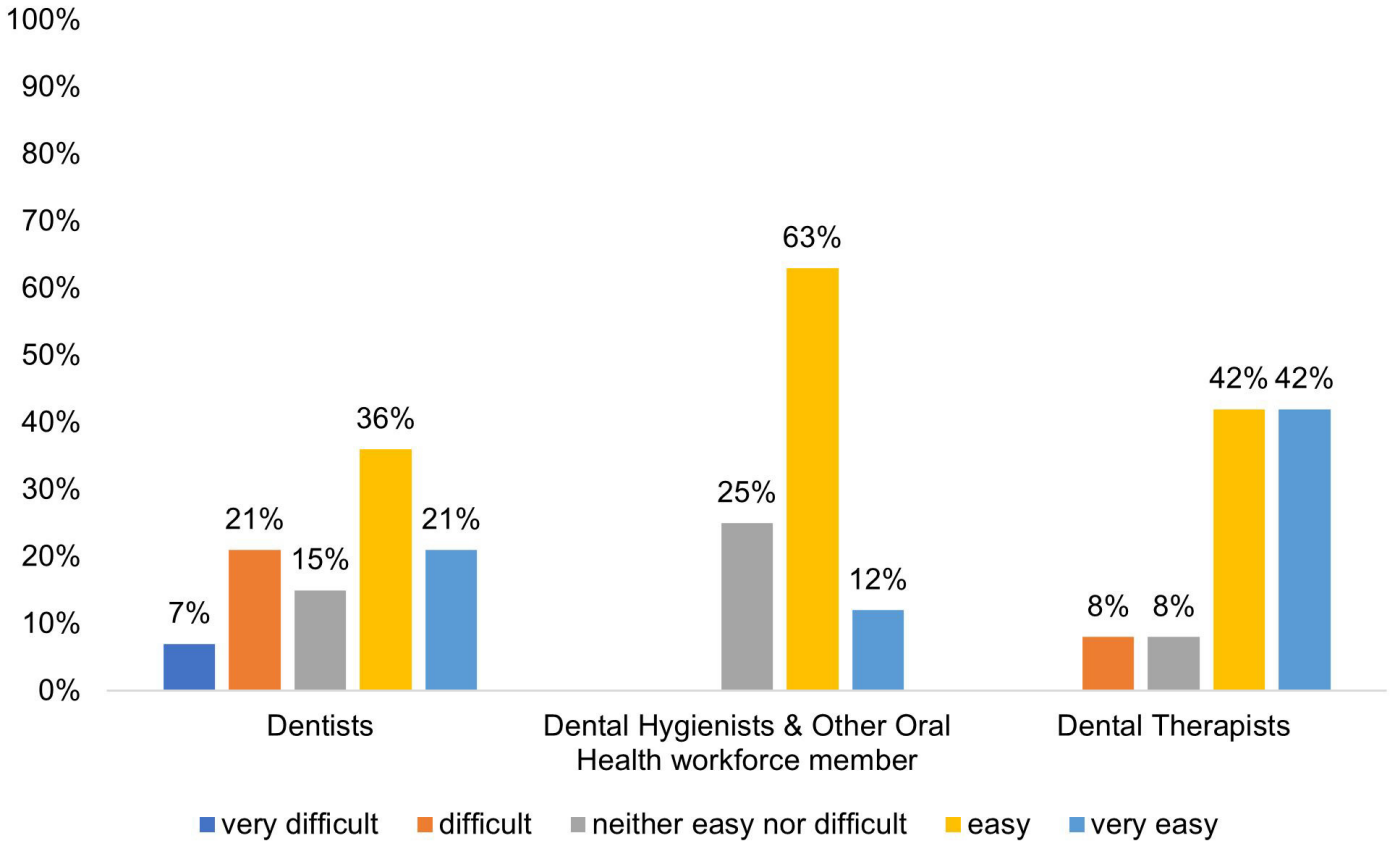
Feelings on incorporating the strategies covered in the ADTA conference into their care of patients and the running of their practice/healthcare clinic, *N* = 56.

Attendees who completed the post conference survey evaluation shared sentiment about incorporating DT into their practice and/or clinic (Figure 1). The majority felt the strategies covered related to DT could be incorporated regardless of the type of workforce position. Most felt the potential to utilize the strategies would be very easy or easy. Fifty-seven percent of dentists felt it would be easy or very easy to implement strategies from the conference, while 28% felt it would be difficult or very difficult. Seventy-five percent of dental hygienists and other oral health workforce members felt it was easy or very easy to implement strategies from the conference while 25% felt it would be neither easy or difficult and none felt it would be difficult or very difficult.

**Figure 2:**
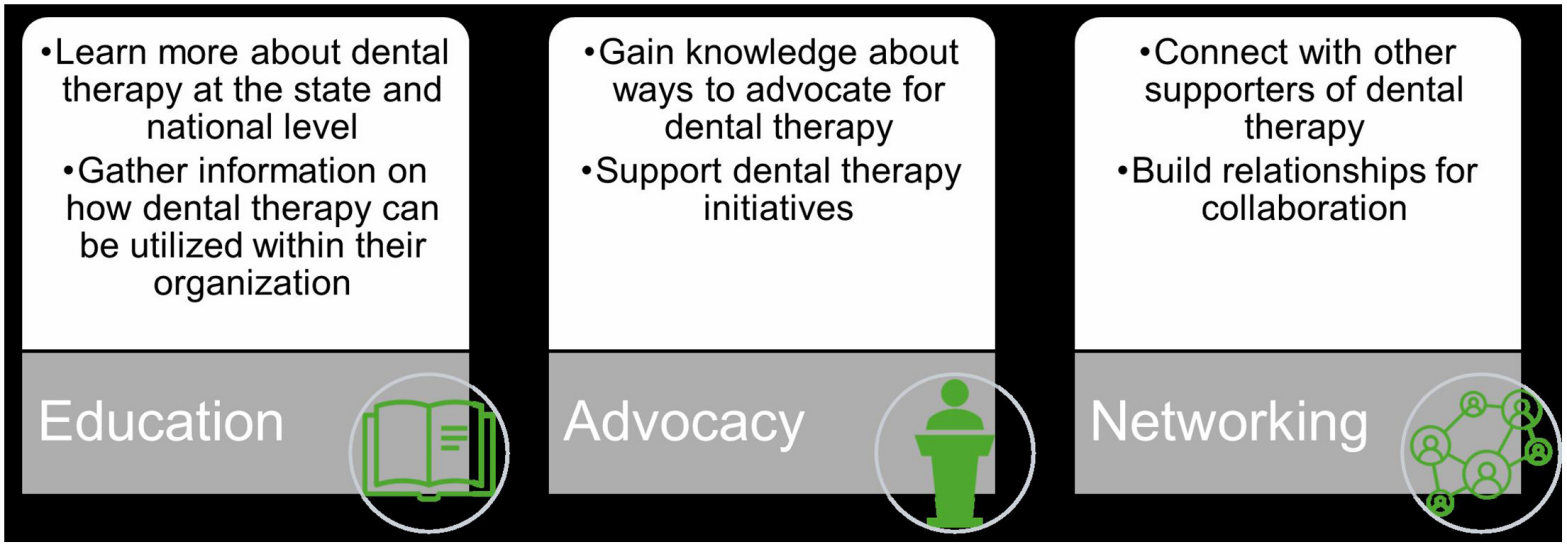
Attendee objectives for joining the ADTA conference, *N* = 56.

To better understand the success of the conference as it relates to promoting awareness of DT, attendees were asked to share their objectives for attending the conference using open-ended statements. These statements were used by researchers to identify three common patterns: education, advocacy, and networking (Figure 2). Attendees shared that they attended the conference because they wanted to learn more about the current state of DT, better understand how it could be incorporated into their practices, understand their role in advocacy for DT, and connect with other supporters of this provider model. These findings suggest that amongst those that completed the survey, attendee objectives aligned with engaging providers on DT.

## Discussion

One hundred people attended the conference from various regions of the U.S. Of the attendees, 56 (56%) completed the post-conference questionnaire. Participants identified as dentists, dental hygienists, dental therapists, other oral health workforce members. Experience in their current roles ranged from 0–26+ years. Regardless of workforce position, most respondents felt the covered DT strategies could be incorporated. Most dentists (57%) felt it would be easy or very easy to implement strategies from the conference, and even more dental hygienists and other oral health workforce members (75%) thought it would be easy or very easy to implement strategies discussed. About 80% of participants responded to open-ended questions exploring personal objectives for attending the conference. Education, advocacy, and networking were identified as main objectives for attending the conference. About 43% provided additional comments or suggestions about the conference. These comments varied but displayed an overall desire for applicable knowledge of DT program logistics and advocacy routes, skill building, and relevant continuing education opportunities.

As legislation continues to sweep across states, the budding profession of DT and expansion of DT programs nationwide is seeing substantial momentum to stretch care and meet community needs. One of the main goals of this conference was to bring together a diverse range of oral healthcare professionals to discuss DT. Efforts were made to invite representatives of Oklahoma Tribes, elected officials, and oral health providers from across the state to listen and learn about the potential implementation of DT in Oklahoma. Attendees nationwide, and locally, participated in the discussions. Comparable to recent findings, attendees generally expressed support for implementing DT in their communities (17). These findings are similar to a recent survey reporting that 58% of respondents want to expand Oklahoma's oral healthcare professions and 96% of respondents support adding the DT professions to help combat oral healthcare inequities (17). Furthermore, these conference data demonstrated a strong inclination for firsthand knowledge, anecdotal experiences, data-driven approaches, and most importantly civil discourse on the topic of mitigating oral health disparities throughout the state and among Tribal communities. The conference platform encourages dissenting viewpoints to contribute to academic and policy debates, and ultimately advance the practice of dentistry by providing broader access to oral healthcare through a comprehensive team approach. Nevertheless, this sample provided nearly unanimous support for the expansion of DT to improve the oral health of, and healthcare available to, AI/AN communities in the lower 48 states of America.

Nearly 30% of dentists felt it would be difficult or very difficult to implement DT strategies covered at the conference while dental hygienists felt neutral to optimistic about potential implementation. The aim of implementing DT providers to the oral healthcare team is to increase timely and affordable access without compromising quality or patient safety to community members who often cannot access oral healthcare (18, 19). This is an adaptable model that allows dentists on the care team to provide complex services to the fullest extent possible with a DT providing appropriate care as needed (18). Though DT is often criticized for lack of safety and efficacy, there are over 100 years of evidence supporting successful DT practice worldwide, and almost 20 years of evidence supporting effective DT practice in the United States (18, 20). Furthermore, no providers chose to comment on this in open-ended responses. Future studies should seek to better understand where opposition from other oral health providers stems from to best establish routes for DT integration into oral healthcare systems.

Though evidence has suggested the success of DT in many communities in the United States (8, 21), identifying the unique needs of the Tribal communities and oral health providers in Oklahoma is important. As previously published from the SPTHB, Tribal citizens and affiliates, Tribal employees, employees of healthcare clinics and agencies, and academics in Oklahoma have expressed interest in incorporating DT into the dental practices they visit, but seek more awareness for community members to better understand the scope of practice of DT (22). The SPTHB and co-chairs brought the ADTA conference to Oklahoma to begin the work of authenticating collaborative partnerships with oral health providers and to understand their perspective on the DT movement. The results from the post-conference survey evaluations indicated that a wide range of oral health professionals attended. In addition to learning more about DT, oral health providers were interested in gathering tools to become advocates of DT authorization and integration. As more states authorize DT, adopt DT policies, and create DT educational programs, Oklahoma should evaluate and leverage the potential of DT for our communities as an essential component of health improvement.

The steps toward authorization of dental therapy in Oklahoma highlight a complex dissonance between the need to expand access to oral healthcare and the challenges of integrating a relatively novel profession into an established oral healthcare delivery system. While dental therapy has years of evidence demonstrating safety, efficacy, and improved access to culturally responsive care, particularly for underserved tribal communities, there remains significant resistance from various vested partners, including portions of the existing dental community, policymakers, and regulatory bodies. This resistance often stems from concerns about quality of care, professional hierarchies, and economic impacts. However, evidence from states where dental therapy has been successfully implemented suggests that these concerns can be mitigated through collaborative approaches, the adoption of the Commission on Dental Accreditation Standards (CODA) for dental therapy education programs, and clear scopes of practice that support community-based care (23).

### Limitations

To our knowledge, this survey is one of few to evaluate oral health provider interest and likelihood of engagement in DT and advocacy, a required step in policy change. Understanding feasibility and frameworks for implementation is a critical step in the direction of further reducing dental healthcare disparities in Tribal and rural Oklahoma communities. Limitations to this study include that the data evaluated included post-conference surveys that were limited to a 56% response rate. There is the potential for selection bias in the post-conference responses indicating a positive reaction to DT because it is often likely that those who attended the conference were already interested in DT. However, efforts were made to invite attendees of all viewpoints, and it was evident from the open-ended statements that learning more about DT was a main objective for attendees. Further, responses come from attendees who are members of the oral health workforce nationwide and may not reflect the viewpoints of those who reside and practice in Oklahoma.

## Conclusion

Oral health providers with diverse scopes of practice support expanding DT to the underserved, rural, and remote communities they serve to improve access to oral healthcare and reduce oral health disparities. Additionally, most providers share sentiment that doing so would be relatively easy, but requires collaborative efforts toward increasing advocacy and knowledge of DT programs. Addressing these oral healthcare inequities and challenges for underserved communities requires a thoughtful, evidence-based approach to improving access to oral healthcare. Collaborative efforts among policymakers, dental associations, educational institutions, and community advocates can create a pathway for the successful integration of dental therapists into the healthcare system.

## Data Availability

The datasets presented in this article are not readily available because the data that support the findings of this study are available from the corresponding author, AL, upon reasonable request. Requests to access the datasets should be directed to amanda.llaneza@mssm.edu.
